# *Amblyomma americanum* as a Bridging Vector for Human Infection with *Francisella tularensis*

**DOI:** 10.1371/journal.pone.0130513

**Published:** 2015-06-29

**Authors:** Rinosh J. Mani, Jessica Abbey Metcalf, Kenneth D. Clinkenbeard

**Affiliations:** 1 Department of Veterinary Pathobiology, Center for Veterinary Health Sciences, Oklahoma State University, Stillwater, Oklahoma, United Sates of America; 2 Oklahoma Animal Disease Diagnostic Laboratory, Stillwater, Oklahoma, United Sates of America; Midwestern University, UNITED STATES

## Abstract

The γ-proteobacterium *Francisella tularensis* causes seasonal tick-transmitted tularemia outbreaks in natural rabbit hosts and incidental infections in humans in the south-central United States. Although *Dermacentor variabilis* is considered a primary vector for *F*. *tularensis*, *Amblyomma americanum* is the most abundant tick species in this endemic region. A systematic study of *F*. *tularensis* colonization of *A*. *americanum* was undertaken to better understand its potential to serve as an overwintering reservoir for *F*. *tularensis* and as a bridging vector for human infections. Colony-reared *A*. *americanum* were artificially fed *F*. *tularensis* subspecies *holarctica* strain LVS via glass capillaries and colonization levels determined. Capillary-fed larva and nymph were initially infected with 10^4^ CFU/tick which declined prior to molting for both stages, but rebounded post-molting in nymphs and persisted in 53% at 10^3^ to 10^8^ CFU/nymph at 168 days post-capillary feeding (longest sampling time in the study). In contrast, only 18% of adults molted from colonized nymphs maintained LVS colonization at 10^1^ to 10^5^ CFU/adult at 168 days post-capillary feeding (longest sampling time). For adults, LVS initially colonized the gut and disseminated to salivary glands by 24 h and had an ID_50_ of <5CFU in mice. *Francisella tularensis* infected the ovaries of gravid females, but transmission to eggs was infrequent and transovarial transmission to hatched larvae was not observed. The prolonged persistence of *F*. *tularensis* in *A*. *americanum* nymphs supports *A*. *americanum* as an overwintering reservoir for *F*. *tularensis* from which seasonal epizootics may originate; however, although the rapid dissemination of *F*. *tularensis* from gut to salivary glands in adults *A*. *americanum* is compatible with intermittent feeding adult males acting as bridging vectors for incidental *F*. *tularensis* infections of humans, acquisition of *F*. *tularensis* by adults may be unlikely based on adult feeding preference for larger mammals which are not involved in maintenance of sylvatic tularemia.

## Introduction

The γ-proteobacterium *Francisella tularensis* subspecies *tularensis* can cause severe systemic infections in natural rabbit hosts and incidental infections in humans in its endemic region in the south-central United States [1–3]. In this endemic region, tularemia is primarily a tick-vectored disease with a spring-summer seasonal pattern corresponding to the questing period of its tick vectors [1]. The American dog tick *Dermacentor variabilis* is thought to be the primary vector for *F*. *tularensis* in its natural rabbit host in the endemic region as well as a possible overwintering reservoir for *F*. *tularensis*, although the lone star tick *Amblyomma americanum* is the most abundant tick species in this endemic region. Infestation of *D*. *variabilis* on small to medium-sized wild mammals has been associated with sylvatic tularemia, and *D*. *variabilis* has been demonstrated to be an experimental vector for *F*. *tularensis* and to support transstadial transmission [1,4–7]. However, the host feeding preference of *A*. *americanum* for larger mammalian hosts including humans may make this tick species more competent vector for transmission of the bacterium to humans [1,8].

*Amblyomma americanum* has been speculated to transmit *F*. *tularensis* in nature and is a competent experimental vector exhibiting transstadial transmission [1,9]. Although *F*. *tularensis* has been detected in wild-caught *A*. *americanum*, its role in transmission of tularemia in nature has not been firmly established [9–11]. To better understand the potential role of *A*. *americanum* in transmission of tularemia, a detailed systematic study of quanta and duration of *F*. *tularensis* colonization of larvae, nymph and adult *A*. *americanum* was undertaken. In addition, the distribution of *F*. *tularensis* in tick tissues of nymph and adults was determined as well as examination of transovarial transmission and transmission to rodents via tick saliva. These studies were accomplished using an artificial colonization method of feeding *F*. *tularensis* to various tick stages using glass capillary tubes placed over the tick mouthparts.

We had previously used this method to study the biology of *F*. *tularensis* in *D*. *variabilis*. In that study, we found that *F*. *tularensis* can infect *D*. *variabilis* as larvae or nymphs and maintain colonization through the adult stage, and infected *D*. *variabilis* can transmit infection to susceptible hosts, making *D*. *variabilis* a potential overwintering reservoir for *F*. *tularensis* [5]. However, the relative high abundance of *A*. *americanum* compared to *D*. *variabilis* in the tularemia endemic region raises the question of what role *A*. *americanum* may play as a vector of *F*. *tularensis* in transmission and persistence of the agent in its endemic region.

Although *D*. *variabilis* and *A*. *americanum* are both ixodid three-host ticks, they differ in many aspects of their lifestyles and natural history which may impact their potential for maintenance and transmission of *F*. *tularensis* in the tularemia endemic region. *Dermacentor variabilis* larvae and nymphs preferentially feed on rodents and medium-sized mammals from which it can acquire *F*. *tularensis*, whereas only the larval stage of *A*. *americanum* feed on rodents or medium-sized mammals from which it might acquire *F*. *tularensis* [12]. These ixodid ticks typically have two to three-year lifespans with *D*. *variabilis* larvae and adults and *A*. *americanum* nymphs surviving the winter in the tularemia endemic region [12–14]. These differences in lifestyles and abundance may result in different roles for these tick species in maintaining and transmitting *F*. *tularensis* in its endemic region, particularly if the biology of *F*. *tularensis* also differs in these ticks.

Although previous studies of *F*. *tularensis* in *A*. *americanum* provided data on infection quanta and demonstrated transstadial transmission [10], our study provides additional detailed systematic studies regarding duration and quanta of colonization as well as examining the tissue localization and transovarial transmission of *F*. *tularensis* in *A*. *americanum*, the latter of which have not previously been reported. For the current study, artificial glass capillary tube feeding method of colonizing ticks with *Francisella* was used to colonize *A*. *americanum* with *F*. *tularensis* subspecies *holarctica* strain LVS. We found that *A*. *americanum* nymphs which acquired *F*. *tularensis* colonization as larvae are able to maintain colonization for sufficient duration and at sufficient quanta to potentially maintain *F*. *tularensis* in the endemic region from one season to the next; however, the feeding preference of *A*. *americanum* nymphs and adults for larger mammals may reduce its importance for maintenance of the tularemia enzootic cycle in rabbits but may facilitate its role as a bridging vector for tularemia in humans.

## Materials and Methods

### Ticks, bacterial strains and growth conditions

*A*. *americanum* larvae, nymphs, and adults were obtained from the Tick Rearing Facility, Department of Entomology and Plant Pathology, Oklahoma State University (Stillwater, OK). Larvae were collected after they were fed to repletion on rabbits. Nymphs were partially fed on sheep until they weighed ≥4.5 mg/nymph. Unfed flat adult ticks were used for quanta and duration of LVS colonization studies and for tissue distribution and infectivity studies. Adult ticks used for salivary induction experiments were partially fed on sheep for five to six days. Female ticks used to study transovarial transmission were fed to repletion on sheep. The tick-feeding on animals were carried out in strict accordance with the recommendations of Institutional Animal Care and Use Committee, Oklahoma State University. The protocol was approved by the Committee (IACUC protocol AG-50-219).

*Francisella tularensis* subsp. *holarctica* strain LVS was supplied by the Oklahoma State Department of Health. A green fluorescent protein (GFP) expressing transformant of LVS was prepared by electroporation of a GFP plasmid (pFNLTP6 *gro-gfp*), gift of Thomas C. Zhart (Medical College Wisconsin, Milwaukee, WI), into LVS [15]. Preparation of inocula and growth of LVS has been described earlier [5]. *Francisella tularensis* was grown on chocolate agar plates (Hardy Diagnostics, Santa Monica, CA.) at 37°C in 5% CO_2_ for 72 h. BBL Prompt Inoculation System (BD Diagnostics, Franklin Lakes, NJ) was used to spike *F*. *tularensis* in the tick inoculum. All chemicals used in the study were purchased from Sigma (St Louis, MO) unless indicated otherwise.

### Capillary tube feeding of larvae, nymphs and adult ticks

The surface disinfection of ticks and the capillary tube-feeding technique have been described earlier [5]. For feeding each batch of ticks, fresh medium was made and spiked with a uniform inoculation dose of 10^7^ CFU/ml LVS. The larvae, nymphs and adults were fed for 18 hours at 30°C and 90% relative humidity. Capillary tube feeding of nymphs and adult ticks was initially assessed by weighing ticks before and after feeding. Only those ticks (nymphs and flat adults) which gained ≥0.3 mg after capillary tube feeding were used for the experiment unless otherwise specified. Based on the estimated minimum tick meal ingested for a ≥0.3 mg body weight gain and the inoculum dose of 10^7^ CFU LVS/ml, the minimum post-capillary feeding infection level is calculated to be 3 × 10^3^ CFU/tick. After feeding, ticks were either surface disinfected by washing for 5 seconds each in 30% hydrogen peroxide, distilled water and 70% isopropyl alcohol and minced for determination of CFUs or were maintained in microcentrifuge tubes capped with moistened cotton plugs and kept in a humidity chamber (relative humidity of > 90%) at 22±1°C (unless specified otherwise) with automated artificial lighting to simulate a 12 h day-night cycle. Determination of CFU per tick was done by sacrificing groups of ticks which were analyzed individually at one day post-capillary feeding and then at an interval of 7 days up to the longest sampling time in these studies of 168 days. To determine the bacterial numbers in ticks at various times of colonization, individual ticks in microcentrifuge tubes were minced with a sterile sharp pointed tweezers, bacteria extracted by incubation in PBS containing 64 μg/mL ampicillin for 2 h at room temperature on a rotor platform mixer (Boekel Scientific, Feasterville, PA.), serially diluted in PBS containing 64 μg/mL ampicillin, and plated on chocolate agar plates for CFU determination following incubation at 37°C in 5% CO_2_ for 72 h. To determine the bacterial numbers in tissues at varying periods of post-capillary tube feeding, individual ticks were dissected into gut, salivary glands, and ovaries under sterile conditions using a dissecting microscope. The tick tissues were placed in PBS containing 64 μg/mL ampicillin and processed as described above for whole ticks. Hemolymph from ticks was collected into sterile glass capillary tubes from the cut ends of the tick’s legs.

### Immunohistochemistry

For immunohistochemical analysis, nymphs which were held in humidity chambers for 40 or 168 days post-capillary tube feeding were fixed in Carson’s fixative, embedded with paraffin and sectioned and affixed to glass slides. After deparaffinizing, the sections were incubated with phosphate buffered saline with 0.05% Tween 20 (PBST) at room temperature for 15 min. and then incubated at 37°C for 1 h with *F*. *tularensis* antiserum (Beckton Dickinson, Sparks, Maryland) at 1/60 dilution in PBST. Antiserum preabsorbed against LVS and uninfected tick sections were used as negative controls (S1 Fig). After washing the slides with PBST five times followed by a final washing with distilled water, the sections were incubated with fluorescein isothiocyanate (FITC) conjugated secondary antibody (KPL, Gaithersburg, MD) at 1/60 dilution in PBST at 37°C for 30 min. The sections were then washed in PBST twice, PBS once and finally washed with distilled water. The slides were visualized using Nikon Eclipse 50i epi-fluorescence microscope and Nikon digital sight DS-5M-L1 digital camera. For visualizing LVS in tick hemolymph, adult ticks were capillary tube fed with LVS expressing GFP. At 1 and 4 weeks post-capillary tube feeding, hemolymph was collected, placed directly on glass slide with coverslip, and visualized using the epi-fluorescent microscope.

### Real-time quantitative PCR

Real- time quantitative PCR was conducted to confirm the absence of LVS in larval and adult ticks and egg masses in which LVS was not detected by microbial culture. Each sample was analyzed using Fast SYBR green master mix on an AB 7500 Fast Real-Time PCR System (Applied Biosystems, Foster City, CA.). A negative control (no template) was processed for each analysis. Samples were assessed to be positive when a 97bp band *F*. *tularensis* ISFtu2 amplification product [16] was confirmed by sequencing and analyzing the dissociation curve. The primer sequence used for the RT-PCR reaction did not detect the presence of *F*. *tularensis* or *Francisella*-like endosymbionts in the colony reared larvae, nymphs or adult ticks prior to feeding ticks LVS. The RT-PCR reaction (20 μL) contained 10 μL Fast SYBR green master mix, 6 μl DNase RNase-free water, 1 μL forward primer (ISFtu2F), 1 μL reverse primer (ISFtu2R) and 2 μL template. Cycling conditions were 95°C for 20 seconds, followed by 34 cycles of 95°C for 10 seconds and 60°C for 30 seconds. Total DNA from each tick (tick minceate in 100 μl PBS) was extracted using DNeasy Tissue Kit (Qiagen, Valencia, CA.), with a final elution volume of 50 μL.

### Infection of ticks by intra-hemocoelic (i.h.) inoculation

To determine the lowest infectious inoculation dose for adult ticks resulting in detection of LVS and to infect gravid females and partially fed adult ticks, 1 μL of the inoculum containing LVS in PBS was injected i.h. in the ventral region of the tick, medial to the caudal most coxa using 10 μL custom-made Hamilton syringe with a 0.5 inch, 33 gauge needle (Hamilton Company, Reno, NV).

### Detection of LVS in tick saliva

To detect LVS in salivary secretion of ticks infected with LVS via capillary tube-feeding, these ticks were induced to secrete saliva by injection with a secretagogue. Two days post-capillary tube feeding, ticks were immobilized dorsal side on the sticky part of duct tape placed on a double-sided adhesive tape. Ticks were then injected i.h. with 4 μL of 1 mM dopamine, 1 mM theophilline and 3% DMSO in PBS (pH 7.3) [17] every 15 min for 1 h. Saliva was collected in a 10 μL (internal diameter of 0.0219 inch) volume glass capillary tubes (Drummond Scientific Company, Broomall, PA) placed over the hypostome of the tick. The capillary tube for collecting saliva was held in place using modeling clay.

### Infective dose 50 in BALB/c mice

The Animal Care and Use Protocol for mouse ID_50_ experiments was approved by the Oklahoma State University Institutional Animal Care and Use Committee (IACUC protocol VM-10-1). To determine infectivity of LVS recovered from tick salivary secretions, eight partially fed adult ticks (colonized with LVS four days previously via capillary tube feeding and held at 27°C) were induced to secrete saliva under sterile conditions, and the saliva from all eight ticks were pooled in 200 μL PBS containing 64 μg/mL ampicillin. The saliva in PBS-ampicillin was diluted to make particular inoculum doses. Five experimental groups of BALB/c mice (six mice in each group) were injected i.p. with 5 CFU, 50 CFU, 500 CFU, 5x10^3^ CFU, and 1x10^4^ CFU LVS from the pooled and diluted saliva. Another five experimental groups of BALB/c mice (six mice in each group) were injected i.p. with 0.4 CFU, 4 CFU, 39 CFU, 194 CFU and 387 CFU of laboratory cultured LVS. One control group of six mice were injected with pooled and diluted tick saliva in PBS-ampicillin from uninfected ticks (capillary tube fed with tick meal without LVS four days previously and held at 27°C). All the mice that showed the clinical endpoint (ruffled haircoat, huddling, lethargy, and decreased mobility) were euthanized. Liver and spleen were aseptically removed form the mice, weighed and homogenized. Blood was collected from the heart immediately after euthanizing the mice, serial 10-fold dilutions made and plated on chocolate agar plates, and CFUs were determined after 72 h of incubation at 37°C and 5% CO_2_. The data from the experiment were used to calculate ID 50 using Reed-Muench method [18].

### Transovarial transmission of LVS in infected gravid adult female *A*. *americanum* to larvae via the egg stage

To assess whether female *A*. *americanum* transovarially transmit *F*. *tularensis* to hatched larvae, adult females fed on sheep to repletion were inoculated via i.h route one day post-repletion either with 1μL of 10^7^ CFU/mL LVS in PBS or with 1μL PBS alone (control). Eggs oviposited between days 10 to 50 post injection were collected and weighed. These eggs after surface sterilization (washing for 5 seconds each in PBS and 70% isopropyl alcohol) were minced and the lysate were serially diluted in PBS containing 64 μg/mL ampicillin and plated for CFU determination. At the completion of oviposition adult female ticks were surface sterilized and hemolymph was collected. The ticks were dissected and tissues including gut, Malpighian tubules, and ovaries were separated out minced and the lysate were serially diluted in PBS-ampicillin and plated for CFU determination. Subsets of the oviposited eggs were placed in microcentrifuge tubes capped with moistened cotton plugs and kept in a humidity chamber for hatching. The hatched larvae after surface sterilization were minced and the lysate were serially diluted in PBS-ampicillin and plated for CFU determination.

### Statistical analysis

*Francisella tularensis* infection levels in different groups of *A*. *americanum* ticks during adult colonization, transstadial transmission from larva to nymphs, and nymph to adult were compared by using 1-way analysis of variance on log-transformed data followed by pairwise multiple comparison of mean CFU values using Holm-Sidak tests. Overall significance level for Holm-Sidak tests was *P* = 0.05. Student's t-test was performed to determine the statistical difference in the mean CFU/infected tick between molted adult male and female *A*. *americanum*. All statistical analyses were performed with SigmaPlot v11.0 Graphics software (Systat Software Inc., Chicago, IL).

## Results

### LVS infection of *A*. *americanum* larvae and transstadial transmission to nymphs

**Fig 1:**
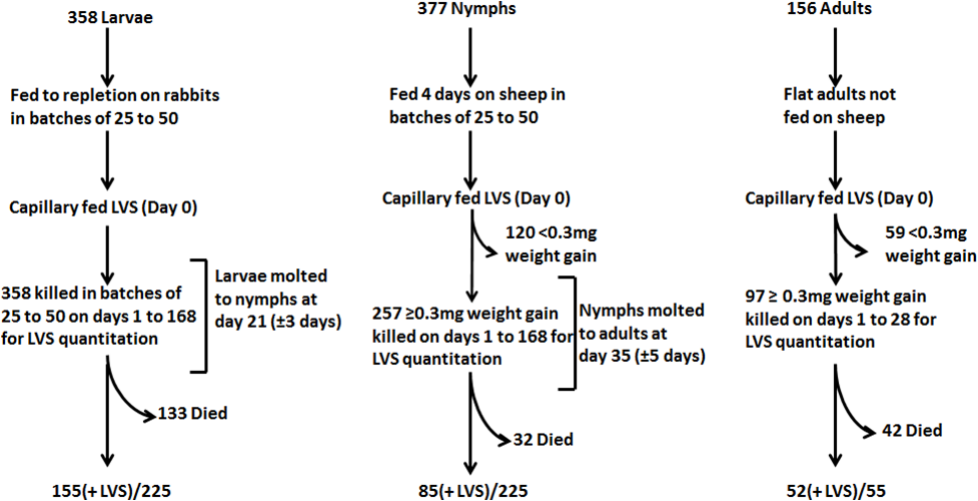
Quantum and duration of colonization and transstadial transmission of LVS experimental design. Larvae, nymphs and adults *A*. *americanum* in batches of various sizes were capillary fed tick meal containing LVS. Ingestion of capillary fed tick meal was determined for nymph and adult ticks by post-feeding weight gains of ≥0.3mg. Nymphs and adults showing <0.3mg weight gains were excluded from further analysis. The overall rate of ticks positive for LVS for each tick life cycle stage capillary tube fed LVS is given as total number of ticks which were positive for LVS (+LVS)/total number of ticks tested.

**Fig 2:**
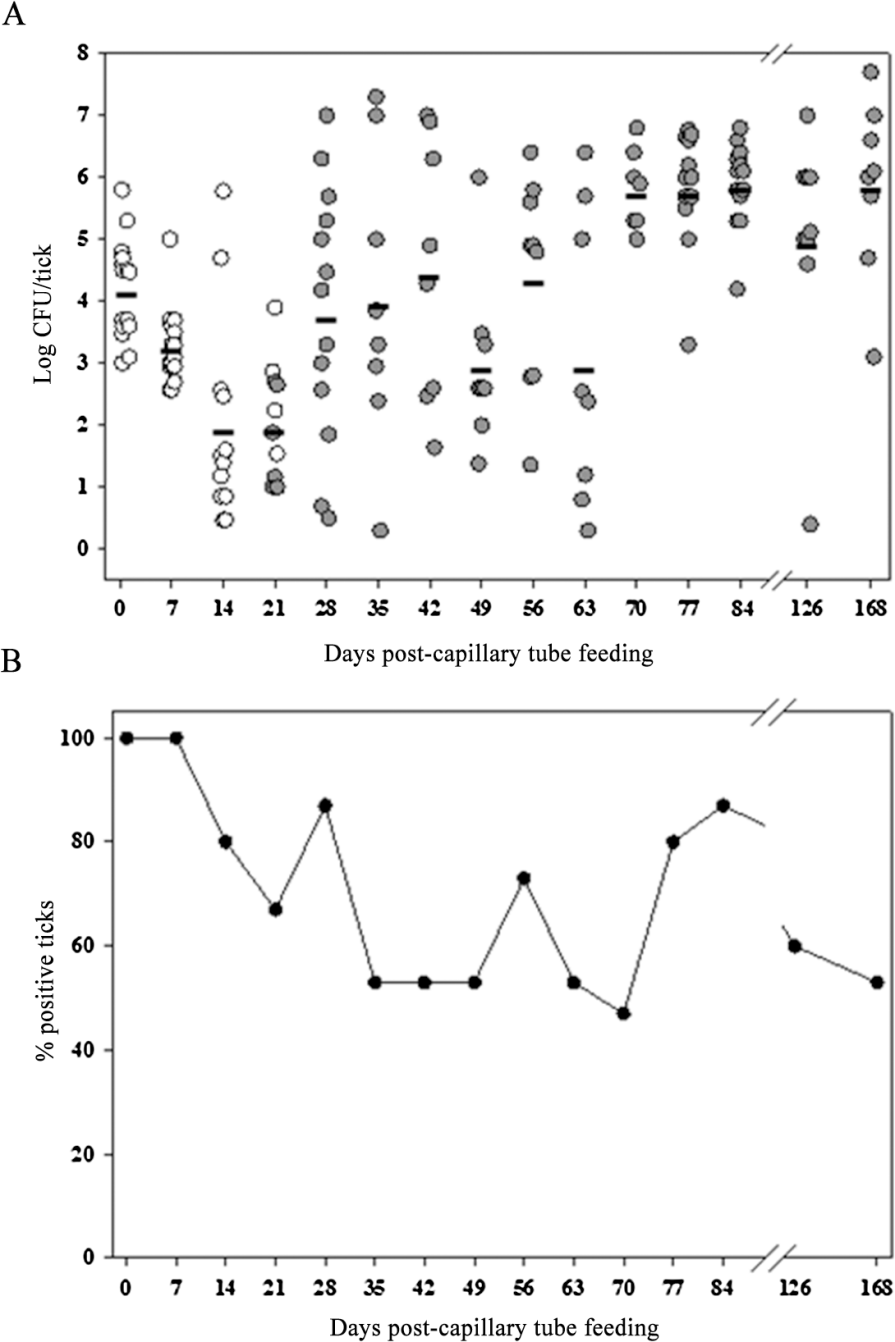
LVS transmitted transstadially from larvae to nymphs. Open circles are capillary tube fed larvae, and filled circles are molted nymphs. The calculated mean CFU/tick for colonized larvae and molted nymphs for each time point is represented by the horizontal line. For each time point, n was 15. (B) Percentage of colonized ticks in the same experiment.

**Fig 3:**
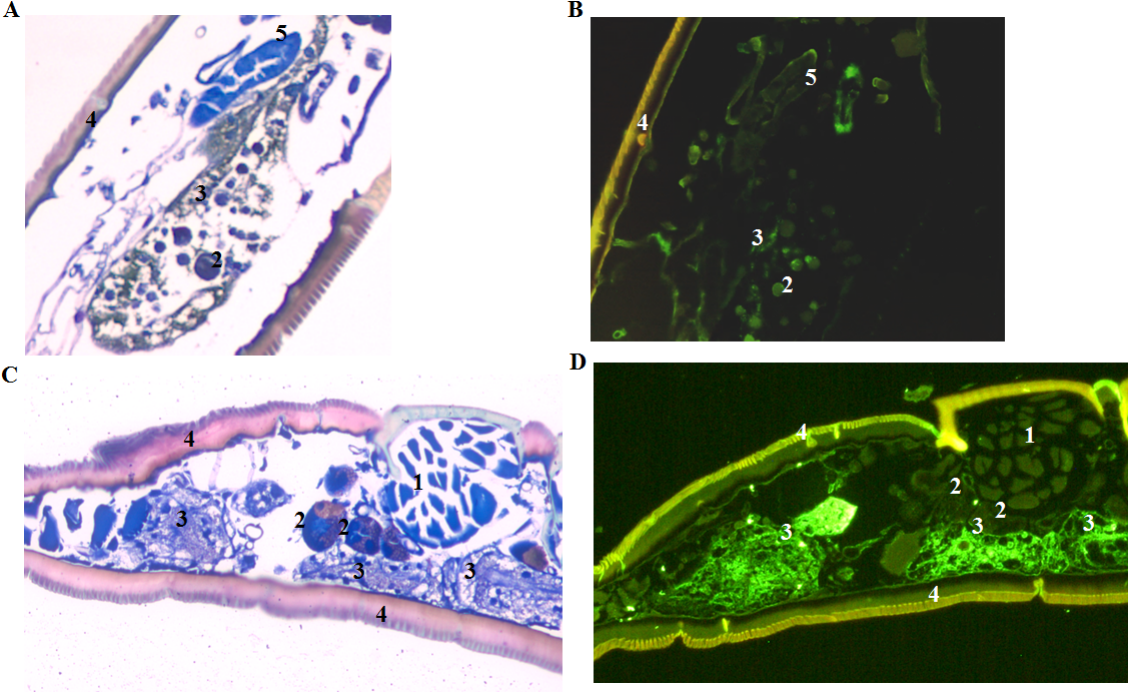
Visualization of LVS in colonized nymphs at 40 and 168 days post-capillary tube feeding. (A) Wright Giemsa stained section of an LVS colonized nymph which was capillary tube fed LVS as a larva, molted to nymph, and was held at 22±1°C for 40 days. (B) Immunostained section of the same nymph. LVS colonization of both the salivary glands and gut was seen. (C) Wright Giemsa stained sections of an LVS colonized nymph which was capillary tube fed LVS as a larva, molted to a nymph, and was held at 22±1°C for 168 days. (D) Immunostained section of the same nymph. LVS colonization was observed only in the gut. Numbers identify (1) muscle, (2) salivary gland, (3) gut, (4) exoskeleton and (5) Malpighian tubule. 200x magnification.

To assess quanta and duration of infection in larval *A*. *americanum* and transstadial transmission of *F*. *tularensis* to nymphs molted from infected larvae, larvae fed to repletion on rabbits were exposed to LVS by capillary feeding tick meal containing 10^7^ CFU/mL LVS. On day one and every seventh day through day 168 post-capillary feeding, groups of larvae or nymphs molted from larvae were assayed individually for LVS (Fig 1). The degree to which larvae took in the capillary fed meal was not ascertainable by weight change in larvae, but at one-day post-capillary tube feeding, LVS was detected with a mean infection level of 1.5 × 10^4^ CFU/larva in 100% of larvae (Fig 2; limit of detection was 2 CFU/larva). For our experimental holding conditions (relative humidity = 95% and mean room temperature of 22±1°C), the larvae molted to nymphs between day 21 and 28 post-capillary tube feeding. Around the time of molting, both the % infected and the mean bacterial count declined (9.6±4.8 × 10^1^CFU/tick, Fig 2). At molting, LVS was detected using immunohistochemistry in gut and in salivary tissue (Fig 3A and 3B). The % infected nymphs remained between 50 and 90% post-molt, but quanta of infection increased to 10^2^ to 10^3^ CFU/nymph between molting and 42 days post-molting, and then increased in level to reach ≈1 × 10^5^ CFU/nymph at 70 days post-capillary feeding (49 days post-molting). This mean level of colonization was maintained through the longest sampling time of 168 days (147 days post-molting) (Fig 2A). Significant difference in the mean colonization levels between molting (day 21) and 70, 77, 84, and 168 days post-capillary feeding (unadjusted *P* < 0.001) was observed. There were no significant differences between any other time points. At 147 days post-molting, LVS was detected only in gut tissue (Fig 3C and 3D). Similar overall survival percentages of 63% and 65% were observed for larvae and nymphs molted from larvae fed LVS compared to those fed tick meal alone.

### LVS infection of *A*. *americanum* nymphs and transstadial transmission to adults

**Fig 4:**
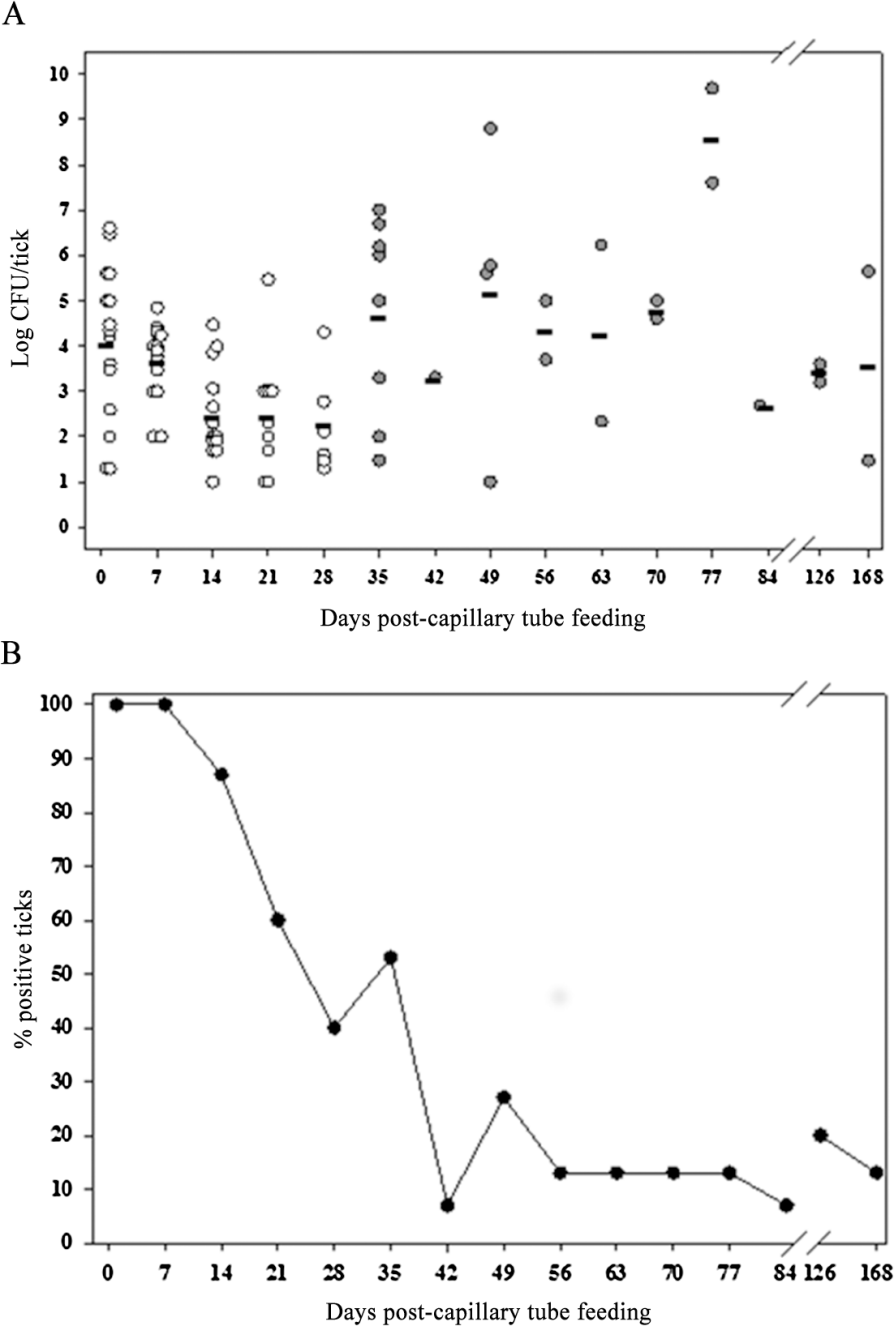
LVS transmitted transstadially from nymphs to adults. (A) Open circles are capillary tube fed nymphs, and filled circles are molted adults. The calculated mean CFU/tick for colonized nymphs and molted adults for each time point is represented by the horizontal line. For each time point up to day 168, n was 15. (B) Percentage of colonized ticks in the same experiment.

To assess quanta and duration of infection *A*. *americanum* nymphs and transstadial transmission of *F*. *tularensis* in *A*. *americanum* adults molted from infected nymphs, nymphs fed for 4 days on sheep were exposed to LVS by capillary feeding tick meal containing 10^7^ CFU/mL LVS. The degree to which nymphs ingested tick meal was ascertained by comparison of pre-feeding and post-feeding weights in which 68% of fed nymph gained ≥0.3 mg/nymph weight gain post-feeding (Fig 1). Sampling only those nymphs with ≥0.3 mg/nymph weight gain post-feeding, on day one LVS was detected in 100% of nymphs with a mean level of 1.3±0.01 × 10^4^ CFU/nymph (Fig 4A). The percent nymphs infected declined towards molting to adults at 35 days, at which time 8/15 adults molted from infected nymphs were positive for *F*. *tularensis* by microbial culture, demonstrating the transstadial transmission of *F*. *tularensis* in capillary tube fed *A*. *americanum* (Fig 4A). There was a progressive decline in the percent of molted adult ticks infected by *F*. *tularensis* to 13% by post-capillary feeding day 56 (day 21 post-molting) and remained at this % at longer sampling times (Fig 4B). Like nymphs molted from infected larvae, the adults molted from infected nymphs showed a decline in quanta of infection until molting, but then the quanta of infection trended higher post-molting (Fig 4A). However there were no significant statistical differences between any time points. The overall survival rate for nymphs and adults molted from nymphs which had increased ≥0.3 mg/nymph weight gain post-feeding was 88% (Fig 1).

### LVS infection of *A*. *americanum* adults

**Fig 5:**
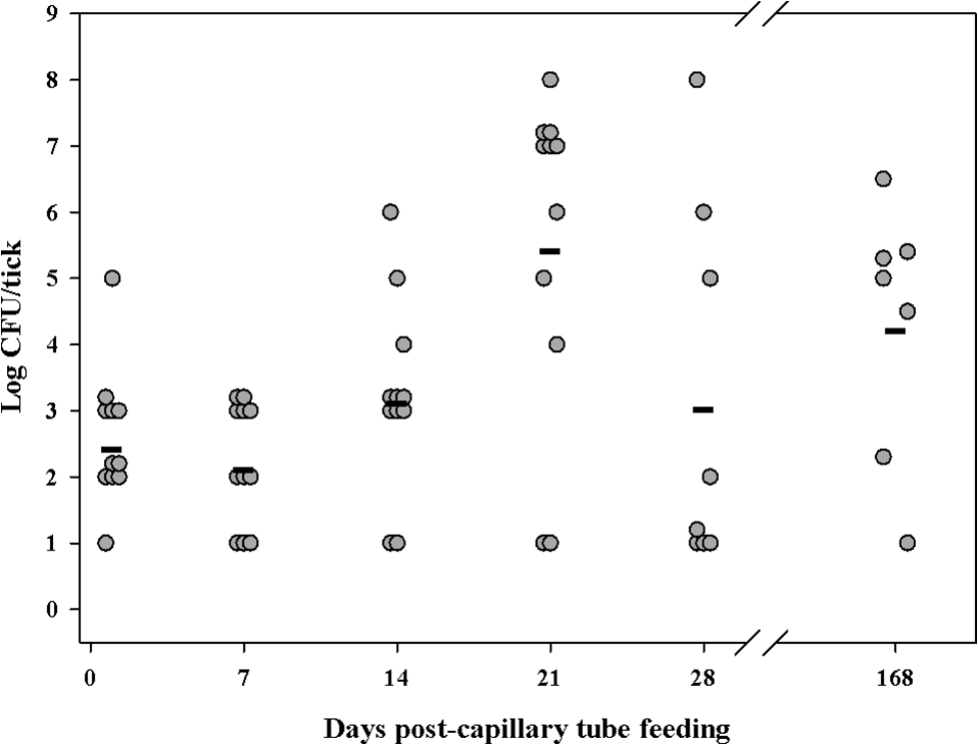
LVS colonization of adult *A*. *americanum*. The filled circles are capillary tube fed adults. The calculated mean CFU/tick for colonized unfed adults for each time point is represented by the horizontal line. For each time point, n was 11; for day 168, n was 29.

To assess quanta and duration of infection of *F*. *tularensis* in *A*. *americanum* adults, flat adults which had not been fed a blood meal as adults were exposed to LVS by capillary feeding tick meal containing 10^7^ CFU/mL LVS. The degree to which adults ingested tick meal was ascertained by comparison of pre-feeding and post-feeding weights in which 62% of fed adults gained ≥0.3 mg/adult weight gain post-feeding (Fig 1). On days one through 28 (Fig 5) and on day 168 post-capillary feeding, groups of adults were assayed for LVS. Sampling only those adults with ≥0.3 mg/tick weight gain post-feeding, a range of mean LVS infection quanta detected were 10^2^ to 10^5^ CFU/tick for the 28 days post-capillary tube feeding period. For the longest sampling time of 168 days post-capillary feeding, 7/29 of flat adults which gained ≥0.3 mg/tick weight gain post-feeding maintained infection with quanta of infection ranging from 10^1^ to 10^6^ CFU/tick. However there were no significant statistical differences between any time points. The overall survival rate for adults which had increased ≥0.3 mg/nymph weight gain post-feeding was 57% (Fig 1).

### Determination of inoculum dose of LVS necessary to establish infection of *A*. *americanum* adults

To determine a minimum infectious dose of *F*. *tularensis* necessary to cause infection of adults ticks, flat adults were injected intra-hemocoelically (i.h.) with 140, 13 or 2 CFUs of LVS. At the high inoculum dose, 10/10 ticks were positive for LVS on day 7 post-injection with a mean quanta of 2.5 × 10^3^ CFU/tick; at a dose of 13 CFU/tick, 8/10 tick were positive on day 7 with a mean quanta of 6.5 × 10^1^ CFU/tick; but for the low inoculum dose none of the ticks were positive for LVS on day 7.

### Tissue localization of LVS in *A*. *americanum* adults

**Fig 6:**
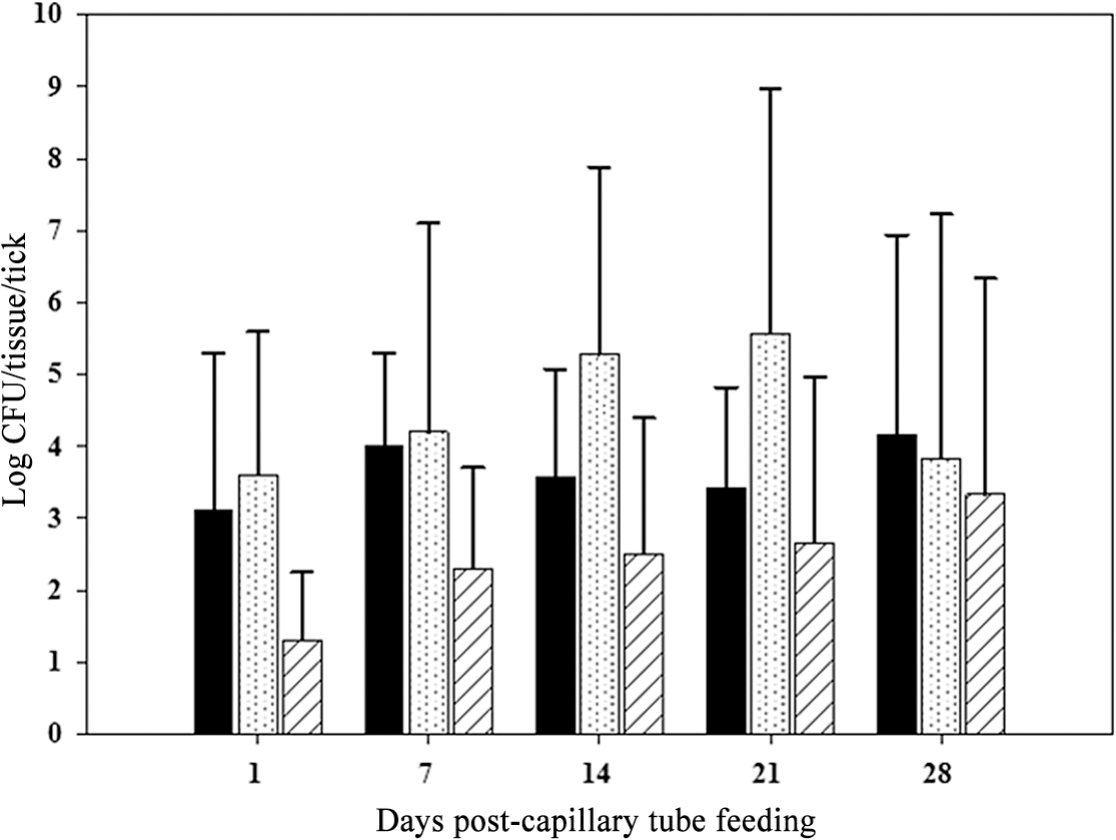
Tissue dissemination of LVS in adult *A*. *americanum*. Solid black bar- gut, white bar with dots- hemolymph, white bar with diagonal lines- salivary gland,. For each time point the n was 5.Error bars indicate standard deviation.

To determine the dissemination of *F*. *tularensis* in tick tissues following infection by ingestion, unfed flat adult ticks were capillary fed tick meal containing 10^7^ CFU/mL LVS and subsequently dissected on various days post-capillary feeding to determine the tissue dissemination of LVS. In adult *A*. *americanum*, LVS penetrated the gut and disseminated to the hemolymph and salivary glands within 24 hours of capillary tube feeding (Fig 6). There were no statistically significant differences in the quanta of infection between the different tissues at any time point.

### LVS secretion in infected tick saliva and infectivity in mice

**Fig 7:**
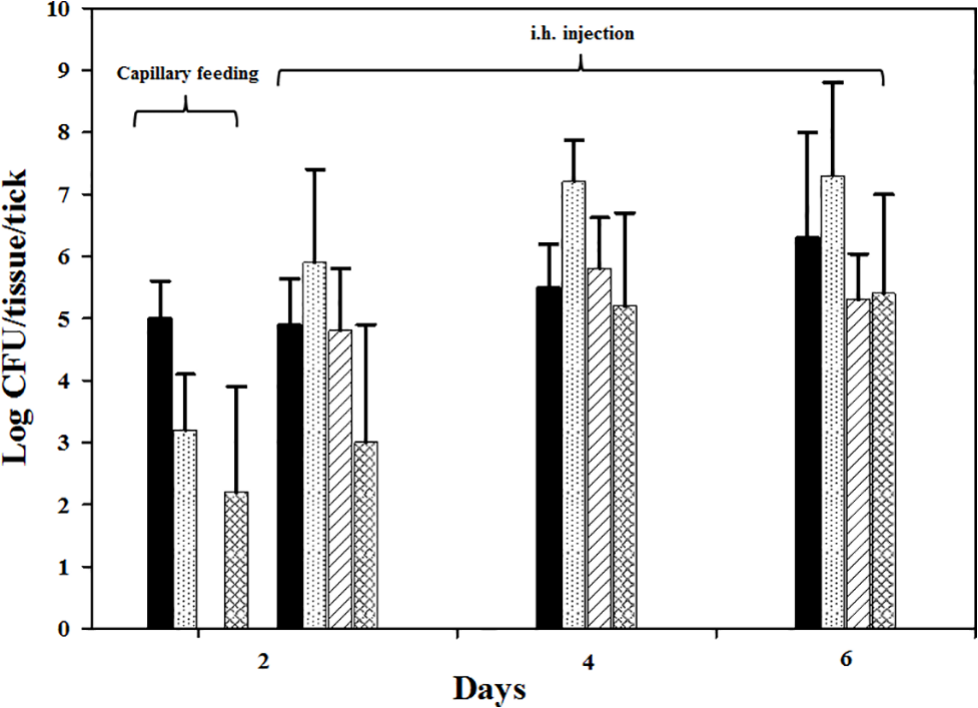
Tissue dissemination of LVS in adult *A*. *americanum* post inoculation. Solid black bar—gut, white bar with dots–hemolymph, white bar with diagonal lines—salivary glands, and white bars with cross marks–saliva (CFU/μl of saliva). For each time point, n was 5. Error bars indicate standard deviation. For day 2 post-capillary tube feeding salivary glands were not collected.

To determine whether LVS disseminated to salivary glands of adult ticks is secreted into saliva and whether these secreted bacteria are infectious, partially fed *A*. *americanum* adults were first inoculated with LVS i.h., and salivary secretion induced by a secretagogue. On day 2 post i.h. inoculation, LVS was detected in gut and salivary gland and was also secreted in the saliva (Fig 7) with a mean level of 1.0±0.1 × 10^3^CFU/μl of saliva. There were no statistically significant differences in the quanta of infection between the different tissues at any time point. In a subsequent experiment, ticks were capillary tube fed with LVS, salivation induced and saliva collected 2 days post-capillary feeding, and saliva pooled, and injected into mice to determine the ID_50_ for LVS in saliva. Similar to what was observed for i.h. inoculation, when ticks were capillary tube fed LVS, the bacterium disseminated from gut to hemolymph and was secreted in the saliva within two days post-capillary tube feeding (Fig 7) with a mean level of 2.0±0.8 × 10^2^CFU/μl of saliva. The ID_50_ for LVS in tick saliva in BALB/c mice injected intra-peritoneally (i.p.) was <5 CFU as compared to 10 CFU for laboratory cultured LVS (Table 1). The mice that had the highest inoculation dose showed clinical signs after 3 days post-injection while the mice that had the lowest inoculation dose showed clinical signs at 5 days post-injection. All mice not showing any clinical signs were euthanized at the end of the study (7 days post injection). Bacterial culture from mouse liver and spleen showed that these tissues were heavily infected with LVS.

**Table 1:** ID_50_ of *F*. *tularensis* LVS from *A*. *americanum* salivary secretions (post-capillary tube feeding) for groups A-E and laboratory cultured LVS for groups F-J in BALB/c mice.

Group	Inoculum (CFUs of LVS)	Fraction of infected mice
A	5	6/6
B	50	6/6
C	500	6/6
D	5000	6/6
E	10000	6/6
F	0.4	0/6
G	4	2/6
H	39	6/6
I	194	6/6
J	387	6/6
Control	Nil^ɑ^	0/6

^ɑ^ Control group of mice was injected with uninfected *A*. *americanum* salivary secretions.

### Transovarial transmission not observed for LVS infected gravid adult female *A*. *americanum* to larvae via the egg stage

To assess whether female *A*. *americanum* transovarially transmit *F*. *tularensis* to hatched larvae, adult females fed on sheep to repletion were inoculated one day post-repletion with LVS i.h., and dissemination of LVS to various tissues and oviposited egg masses assessed along with transovarial transmission to hatched larvae. After dissection of the infected gravid ticks at completion of oviposition, *F*. *tularensis* was detected in hemolymph, gut, Malpighian tubules, and ovaries (data not shown); but only 5/18 egg masses oviposited between days 10 and 50 post-injection were positive for *F*. *tularensis* by microbial culture (range 0.08 to 9.0 CFUs/mg egg mass). Transovarial transmission of *F*. *tularensis* to hatched larvae was not detected by either microbial culture or RT-qPCR for larvae hatched from infected and non-infected egg masses.

## Discussion

The persistence of tick borne *F*. *tularensis* subspecies *tularensis* in the south-central United States is perpetuated by a tick-small mammalian host enzootic cycle of sylvatic tularemia [8,19,20]. Historically, *D*. *variabilis* has been considered the primary vector for *F*. *tularensis* in this endemic region [1]. However, the most abundant tick species found in this region is *A*. *americanum*, which consistently accounted for more than 90% of the total ticks in a number of tick surveys done in this region [8,9,21,22]. The host questing seasonality as well as the host feeding preference likely determines the role *A*. *americanum* may play in maintenance of the enzootic tularemia cycle as well as its potential for bridging infections to incidental hosts such as humans. In addition, the capacity of *F*. *tularensis* to maintain colonization of tick salivary glands is another factor determining the roles these tick may play as vectors for tularemia. We report herein a study of the capacity of *F*. *tularensis* to maintain colonization in *A*. *americanum* and assess the potential for *A*. *americanum* to serve as a vector of *F*. *tularensis* based on colonization capacity and natural history.

The only previous quantitative studies of *F*. *tularensis* persistence in *A*. *americanum* known to the authors were published by Hopla in 1953 and 1955. In the 1955 study, Hopla sought to characterize persistence and multiplication of *F*. *tularensis* in various stages of *A*. *americanum* infected by acquisition feeding as ticks as larvae. Our study differs from the Hopla study in that *A*. *americanum* larvae, nymphs and adults were infected with *F*. *tularensis* by artificial capillary tube feeding. An advantage of acquisition feeding studies like Hopla’s are that ticks receive physiologic cues while feeding potentially important for both the tick vector and the bacterium that are likely missing in capillary tube feeding infection of ticks such as our experiments. However, using capillary tube feeding to infect ticks, relatively large numbers of ticks can be infected by exposure to an inocula of standardized bacterial suspension, whereas with acquisition feeding, the duration and intensity of the bacteremia in *F*. *tularensis* infected laboratory animals is short and variable, respectively, making it difficult to standardize tick exposure to the agent. A recent report confirmed the difficult of acquisition feeding for infection of *A*. *americanum* with LVS [7]. In particular, these researchers observed that acquisition feeding with *A*. *americanum* on LVS infected mice was more variable and prolong than similar acquisition feeding with *D*. *variabilis*, making acquisition feeding for LVS infection of *A*. *americanum* less practical for generating sufficient numbers of infected ticks for study.

A second difference of our study and Hopla’s was the subspecies of *F*. *tularensis* used. Hopla used *Bacterium tularense* strain Sm [23], which was likely a fully virulent *F*. *tularensis* subspecies *tularensis*, the original of which we cannot identify, whereas we used an attenuated subspecies *holarctica* strain which is extensively characterized. It is not known whether attenuated *F*. *tularensis* strains such as LVS behave differently in tick vectors than wild-type strains, but a comparison of our study with that of Hopla’s may be instructive.

Of particular interest for our study is the potential for *A*. *americanum* to serve as an overwintering reservoir for *F*. *tularensis* in its sylvatic cycle by maintenance of colonization in nymphs which constitute the primary *A*. *americanum* stage that survives overwintering in the south-central U.S. endemic region [12]. From our study, *A*. *americanum* infected as larvae exhibited potential to maintain prolonged colonization as unfed nymphs compatible with overwintering. This observation is consistent with Hopla’s earlier observations in which he infected larvae and determined their quanta of infection through the adult stage by subsequent feeding on uninfected guinea pigs to molt infect larvae to nymphs and adults [23]. Hopla reported similar quanta of infection in engorged larvae and unfed nymphs to quanta that we observed in 1 day post-capillary feeding larvae and 14 day post-molting nymph of log 4 and 5, respectively. Hopla refed these unfed nymphs molted from infected larvae immediately following molting such that there is no comparable data for persistence of *F*. *tularensis* unfed nymphs in his study to assess potential for overwintering of the agent in unfed nymphs. From our study, we observed that *F*. *tularensis* persisted in unfed *A*. *americanum* nymphs for up to 168 days post-capillary feeding (147 days post molting), which was the longest sampling time. However, the tissue distribution of *F*. *tularensis* in unfed *A*. *americanum* nymphs changed from gut and salivary glands at 19 days after molting to primarily gut at 147 days post-molt. It would appear that *F*. *tularensis* would have to re-disseminate to salivary glands for the nymphs to be infective for initiating the next seasonal cycle. There is some evidence from Hopla’s studies and for other tick and pathogens that refeeding does elicit pathogen secretion [23–26].

While our observations for quanta of infected larvae and unfed nymphs molted from infected larvae are similar to those of Hopla, our observation for infect nymphs and adults molted from infect nymphs differ from Hopla’s infected larvae molted to nymphs and subsequently to adults [23]. In Hopla’s experiments, the infection quanta reached log 9, the highest level observed at any stage, for nymphs prior to molting to adults, but then declined to log 6.5 for unfed adults at 5 weeks post-molting. In contrast, at 21 days post-capillary feeding infection of nymphs corresponding to Hopla’s “prior to molting” stage, nymphs in our experiment exhibited a decline in infection quanta to a nadir of log 2 before rebounding at 5 weeks post-molting as unfed adults to log 4. Pre-molting nadirs of infection levels have previously been observed by us for *F*. *tularensis* in *D*. *variabilis* as well as by others for *Borrelia burgdorferi* in *Ixodes dammini* [5,27]. It is assumed that high levels of bacterial CFUs/ticks correlates with active infection and superior potential for vector transmission; however, the relationship between infection level and vector competency is not known with any specificity for tularemia.

In adult *A*. *americanum* infected as nymphs by capillary feeding, we observed that *F*. *tularensis* maintained infection in only 10% of ticks for a period up to 140 day post-molting. This is in contrast to Hopla’s observations for adult ticks infected with *F*. *tularensis* via acquisition feeding as larvae retained infection through molts to adults with 79% of these adult ticks being positive for *F*. *tularensis* at 150 day post-molting [23]. Based on these observations, *A*. *americanum* nymphs or adults acquiring infection as either larvae or nymphs could act as enzootic vectors for natural sylvatic hosts or as bridging vectors for *F*. *tularensis* of incidental hosts such as humans during spring-summer host seeking periods. In particular, the host feeding preference of nymph and adult *A*. *americanum* for larger mammalian species such as humans would be expected to facilitate it as a bridging vector for *F*. *tularensis* in humans.

As mentioned earlier for nymphs, an additional consideration for *A*. *americanum* serving as a vector of *F*. *tularensis* is secretion of the agent in saliva. For *A*. *americanum* infected as adults, *F*. *tularensis* penetrated the gut and reached the salivary glands as early as 24 hours post-capillary tube feeding in adult *A*. *americanum*. This is in contrast to our earlier report for *D*. *variabilis*, in which *F*. *tularensis* required 2 to 3 weeks to penetrate gut and reach the hemolymph and salivary glands [5]. The rapid dissemination of *F*. *tularensis* in *A*. *americanum* has implications regarding the vector competence of this tick. The extrinsic incubation period, demonstrated by the presence of *F*. *tularensis* in the saliva of the infected *A*. *americanum* adult ticks was found to be as low as 48 hours post-capillary tube feeding. This short extrinsic incubation period may have limited significance for transmission of *F*. *tularensis* by adult female *A*. *americanum* which are committed feeders, but adult male *A*. *americanum* are intermittent feeders such that adult male which acquire *F*. *tularensis* from an infected host can drop off and infest a second host and transmit *F*. *tularensis* to this new host [14], similar to the role of intermittent feeding by adult male *Dermacentor andersoni* ticks for the transmission of *Anaplasma marginale* in cattle [28,29]. However, the likelihood of adult male *A*. *americanum* acquiring *F*. *tularensis* based on their preferential feeding on larger host makes subsequent intermittent feeding transmission of *F*. *tularensis* to another large incident host such as humans appear problematic.

All three stages of *A*. *americanum* including adults, nymphs, and larvae have been found naturally infected with *F*. *tularensis* in nature [9,11,22]. The presence of *F*. *tularensis* in unfed larvae raised the possibility of transovarial route of transmission in this tick species. We examined the possible transovarial transmission of *F*. *tularensis* in *A*. *americanum* and found that occasional transmission to eggs could occur although with a relatively low level of *F*. *tularensis* infection per egg mass. However, the transmission of *F*. *tularensis* from eggs to hatched larvae was not observed. Despite the detection of *F*. *tularensis* in unfed *A*. *americanum* larvae in nature, the potential role of transovarial transmission of *F*. *tularensis* from infected adult females to hatched larvae in maintenance of *F*. *tularensis* infections in *A*. *americanum* appears unlikely. The lack of finding of large number of infected larvae in nature also supports the conclusion reached by previous researchers that transovarial transmission of *F*. *tularensis* in ticks is the exception rather than the rule [4,30].

Although extensively studied, the reservoir host for *F*. *tularensis* is still questionable in the South-Central United State endemic region [31]. The high mortality rate for tularemia in lagomorphs makes status of this species as a reservoir of *F*. *tularensis* uncertain [31]. Rodents are another obvious candidate as a reservoir host for tularemia, but there is currently insufficient data to support their role as a reservoir. Ticks are the third species in the south-central United States endemic region which may maintain *F*. *tularensis* between seasonal outbreaks. The data presented herein demonstrates the potential for *A*. *americanum* nymphs which acquire *F*. *tularensis* as larvae to maintain the agent, overwinter and to initiate new seasonal outbreaks via a tick-small mammalian life cycle. Additional studies are needed to address the issue of whether rabbits, rodents or ticks serve as reservoirs for *F*. *tularensis* in its endemic regions [20,32,33]. Based on Hopla’s and our studies, nymph or adult *A*. *americanum* which acquired *F*. *tularensis* as larvae or nymphs may serve as bridging vectors by recrudescence of *F*. *tularensis* colonization from tick gut to salivary glands during feeding to initiate subsequent seasonal tularemia epizootics.

## Supporting Information

S1 Fig Specificity of the immunohistochemical staining. A and B- Immunostained sections of nymphs fed with LVS at 40 day post-capillary tube feeding (adsorbed serum used as primary antibody). C and D- Immunostained sections of unfed ticks using *F*. *tularensis* antiserum as primary antibody. 200x magnification.
(TIFF)

## References

[1] Eisen L. A call for renewed research on tick-borne *Francisella tularensis* in the Arkansas-Missouri primary national focus of tularemia in humans. J Med Entomol. 2007; 44: 389–397. 17547223 10.1603/0022-2585(2007)44[389:acfrro]2.0.co;2

[2] Goodman JL, Dennis DT, Sonenshine DE. Tick-Borne Diseases of Humans. Washington, D.C.: ASM press; 2005.

[3] Jellison WL. TULAREMIA in North America. Missoula, Montana: University of Montana; 1974.

[4] Bell JF. Infection of ticks (*Dermacentor variabilis*) with *Pasteurella tularensis* . J Infect Dis. 1945;76: 83–95.

[5] Mani RJ, Reichard MV, Morton RJ, Kocan KM, Clinkenbeard KD. Biology of *Francisella tularensis* Subspecies *holarctica* Live Vaccine Strain in the Tick Vector *Dermacentor variabilis* . PLoS One. 2012;7: e35441 10.1371/journal.pone.0035441 22530023 PMC3329428

[6] Philip CB, Jellison WL. The American Dog Tick, *Dermacentor variabilis* as a Host of *Bacterium tularense* . Public Health Rep. 1934; 49: 386–392.

[7] Coburn J, Maier T, Casey M, Padmore L, Sato H, Frank DW. Reproducible and Quantitative Model of Infection of *Dermacentor variabilis* with the Live Vaccine Strain of *Francisella tularensis* . Appl Environ Microbiol. 2015;81: 386–395. 10.1128/AEM.02917-14 25362054 PMC4272728

[8] Hopla CE. The transmission of tularemia organisms by ticks in the southern states. South Med J. 1960; 53: 92–97. 14403044 10.1097/00007611-196001000-00020

[9] Calhoun EL. Natural occurrence of tularemia in the lone star tick, *Amblyomma americanus* (Linn.), and in dogs in Arkansas. Am J Trop Med Hyg. 1954; 3: 360–366. 13138839 10.4269/ajtmh.1954.3.360

[10] Hopla CE. Experimental studies on tick transmission of tularemia organisms. Am J Hyg. 1953; 58: 101–118. 13065276 10.1093/oxfordjournals.aje.a119585

[11] Hopla CE, Downs CM. The isolation of *Bacterium tularese* from the tick *Amblyomma americanum* . J Kansas Entomol. 1953; Soc 26: 72–73.

[12] Kollars TM Jr, Oliver JH Jr, Durden LA, Kollars PG. Host association and seasonal activity of *Amblyomma americanum* (Acari: Ixodidae) in Missouri. J Parasitol. 2000; 86: 1156–1159. 11128501 10.1645/0022-3395(2000)086[1156:HAASAO]2.0.CO;2

[13] Kollars TM Jr, Oliver JH Jr, Masters EJ, Kollars PG, Durden LA. Host utilization and seasonal occurrence of *Dermacentor* species (Acari:Ixodidae) in Missouri, USA. Exp Appl Acarol. 2000; 24: 631–643. 11201355 10.1023/a:1026566301325

[14] Sonenshine DE. Biology of Ticks. New York: Oxford University Press; 1991.

[15] Maier TM, Havig A, Casey M, Nano FE, Frank DW, Zahrt TC. Construction and characterization of a highly efficient *Francisella* shuttle plasmid. Appl Environ Microbiol. 2004; 70: 7511–7519. 15574954 10.1128/AEM.70.12.7511-7519.2004 PMC535190

[16] Versage JL, Severin DD, Chu MC, Petersen JM. Development of a multitarget real-time TaqMan PCR assay for enhanced detection of *Francisella tularensis* in complex specimens. J Clin Microbiol. 2003; 41: 5492–5499. 14662930 10.1128/JCM.41.12.5492-5499.2003 PMC309004

[17] Jaworski DC, Simmen FA, Lamoreaux W, Coons LB, Muller MT, Needham GR. A secreted calreticulin protein in ixodid tick (*Amblyomma americanum*) saliva J Insect Physiol. 1995; 41: 369–375.

[18] Lennette EH. General principles underlying laboratory diagnosis of viral and rickettsial infections; Lennette EH, Schmidt NJ, editors. New York: American Public Health Association Inc; 1964.

[19] Assal NR, Lindeman RD, Carpenter RL. Epidemiologic study on reported human tularemia in Oklahoma, 1944–65. J Okla State Med Assoc. 1968; 61: 120–124. 5641655

[20] Petersen JM, Mead PS, Schriefer ME. *Francisella tularensis*: an arthropod-borne pathogen. Vet Res. 2009; 40: 7 10.1051/vetres:2008045 18950590 PMC2695023

[21] Brown HE, Yates KF, Dietrich G, Macmillan K, Graham CB, Reese SM, et al An acarologic survey and *Amblyomma americanum* distribution map with implications for tularemia risk in Missouri. Am J Trop Med Hyg. 2011; 84: 411–419. 10.4269/ajtmh.2011.10-0593 21363979 PMC3042817

[22] Calhoun EL, Alford HI Jr. Incidence of tularemia and Rocky Mountain spotted fever among common ticks of Arkansas. Am J Trop Med Hyg. 1955; 4: 310–317. 14361910 10.4269/ajtmh.1955.4.310

[23] Hopla CE. The multiplication of tularemia organisms in the lone star tick. Am J Hyg. 1955; 61: 371–380. 14376386 10.1093/oxfordjournals.aje.a119761

[24] Piesman J, Mather TN, Sinsky RJ, Spielman A. Duration of tick attachment and *Borrelia burgdorferi* transmission. J Clin Microbiol. 1987; 25: 557–558. 3571459 10.1128/jcm.25.3.557-558.1987 PMC265989

[25] Spencer RR, Parker RR. Rocky Mountain spotted fever: infectivity of fasting and recently fed ticks. Public Health Reports. 1923; 38: 333–339. 19314866

[26] Katavolos P, Armstrong PM, Dawson JE, Telford SR III. Duration of tick attachment required for transmission of granulocytic ehrlichiosis. J Infect Dis. 1998; 177: 1422–1425. 9593039 10.1086/517829

[27] Piesman J, Oliver JR, Sinsky RJ. Growth kinetics of the Lyme disease spirochete (*Borrelia burgdorferi*) in vector ticks (*Ixodes dammini*). Am J Trop Med Hyg. 1990; 42: 352–357. 2331043 10.4269/ajtmh.1990.42.352

[28] Kocan KM, Stiller D, Goff WL, Claypool PL, Edwards W, Ewing SA, et al Development of *Anaplasma marginale* in male *Dermacentor andersoni* transferred from parasitemic to susceptible cattle. Am J Vet. 1992; Res 53: 499–507. 1586018

[29] Kocan KM, Goff WL, Stiller D, Edwards W, Ewing SA, Claypool PL, et al Development of *Anaplasma marginale* in salivary glands of male *Dermacentor andersoni* . Am J Vet Res. 1993; 54: 107–112. 8427453

[30] Hopla CE. The ecology of tularemia. Adv Vet Sci Comp Med. 1974; 18: 25–53. 4419176

[31] McCahan GR, Moody MD, Hayes FA. An epizootic of tularemia among rabbits in northwestern South Carolina. American Journal of Epidemiology. 1962; 75: 335–338.

[32] Petersen JM, Molins CR. Subpopulations of *Francisella tularensis* ssp. tularensis and *holarctica*: identification and associated epidemiology. Future Microbiol. 2010; 5: 649–661. 10.2217/fmb.10.17 20353304

[33] Kugeler KJ, Mead PS, Janusz AM, Staples JE, Kubota KA, Chalcraft LG, et al Molecular epidemiology of *Francisella tularensis* in the United States. Clin Infect Dis. 2009; 48: 863–870. 10.1086/597261 19245342

