# Supplementary figures and images for: Amblyomma americanum as a Bridging Vector for Human Infection with Francisella tularensis
Source: PLoS One. 2015 Jun 29;10(6):e0130513. doi: 10.1371/journal.pone.0130513 (PMC4486451; doi:10.1371/journal.pone.0130513)

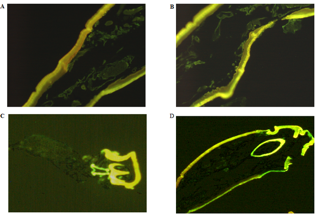

Supplement: S1 Fig — A and B- Immunostained sections of nymphs fed with LVS at 40 day post-capillary tube feeding (adsorbed serum used as primary antibody). C and D- Immunostained sections of unfed ticks using F. tularensis antiserum as primary antibody. 200x magnification. (TIFF) [file pone.0130513.s001.tiff]
